# Big Data Analytics and the Struggle for Equity in Health Care: The Promise and Perils

**DOI:** 10.1089/heq.2019.0112

**Published:** 2020-04-01

**Authors:** Said A. Ibrahim, Mary E. Charlson, Daniel B. Neill

**Affiliations:** ^1^ Division of Healthcare Delivery Science & Innovation, Weill Cornell Medicine, New York, New York, USA.; ^2^ Department of Population Health Sciences, Weill Cornell Medicine, New York, New York, USA.; ^3^ The William T. Foley Distinguished Professor in Medicine, Weill Cornell Medicine, New York, New York, USA.; ^4^ Department of Medicine, Weill Cornell Medicine, New York, New York, USA.; ^5^ Center for Urban Science and Progress, New York University, New York, New York, USA.; ^6^ Courant Institute of Mathematical Sciences, Department of Computer Science, New York University, New York, New York, USA.; ^7^ Robert F. Wagner Graduate School of Public Service, New York University, New York, New York, USA.

**Keywords:** health care innovation, health care disparities, big data analytics, algorithmic decision-making, inequities, biases

## Abstract

Big data is both a product and a function of technology and the ever-growing analytic and computational power. The potential impact of big data in health care innovation cannot be ignored. The technology-mediated transformative potential of big data is taking place within the context of historical inequities in health and health care. Although big data analytics, properly applied, hold great potential to target inequities and reduce disparities, we believe that the realization of this potential requires us to explicitly address concerns of fairness, equity, and transparency in the development of big data tools. To mitigate potential sources of bias and inequity in algorithmic decision-making, a multipronged and interdisciplinary approach is required, combining insights from data scientists and domain experts to design algorithmic decision-making approaches that explicitly account and correct for these issues.

Despite the potential of big data analytics to transform health care, the jury is still out on how they will impact equity in utilization and outcomes of health care.^1^ At the population level, health care disparities cost an estimated $309 billion annually. The difference in life expectancy between the most privileged members of our society and those from disadvantaged backgrounds is about 15 years. Income, a strong correlate of race/ethnicity in the United States, accounts for almost a third of excess mortality among African Americans compared with whites.^2^

Many recent and emerging health care innovations involve big data. Although big data holds great potential to target inequities and reduce disparities, machine learning algorithms generated from big data also have the potential to exacerbate existing disparities and create new ones. To minimize this risk, data must be representative of the population at large and the benefits it confers must be available to all. The digital divide is a threat to this ideal. Social determinants of health still shape access to technology. About 11% of U.S. adults do not use the Internet. Low-income communities and households are about four times more likely than middle- or high-income communities to lack access to broadband technology. Similarly, 21% of uninsured patients do not use the Internet and a much larger percentage of patients do not seek health information online. Thus, differential access to technology not only threatens the representativeness of the data that populate our big data models and inform the resultant algorithms, but also undermines the potential of big data to improve the lives of the most vulnerable people.^3,4^

Furthermore, big data techniques such as machine learning and artificial intelligence may not reflect the diversity of perspectives and backgrounds needed to assure fairness and reduce bias in the algorithms they create. Evidence in the nonhealth care sector suggests that demographic and socioeconomic disparities can arise from targeted advertising or price discrimination. It is well known that cigarette manufacturers target advertisements to low-income and minority neighborhoods. Perhaps less well known, online retailers may vary prices and deals based on users' geographic and demographic information.^5^ Although the financial motivation may be to better manage supply and demand, one consequence is that lower store density in poor and ethnic minority neighborhoods entails higher prices in these neighborhoods, contributing to disparities.

The use of predictive tools such as Northpointe's Correctional Offender Management Profiling for Alternative Sanctions (COMPAS) software in criminal justice to inform sentencing and parole decisions by predicting individual's' risk of reoffending offers another cautionary example. An analysis of COMPAS predictions and subsequent rearrests in Broward County, Florida, by the NGO ProPublica concluded that COMPAS was biased against African American defendants, although Northpointe has disputed these conclusions. Our reanalysis of the COMPAS data, although not providing strong evidence of systematic racial bias, reveals several other systematic biases in COMPAS predictions, such as underweighting the importance of an individual's number of prior offenses, and overestimating reoffending risk for females who have committed minor offenses.

The reasons for these algorithm-based biases are varied, but they can have a profound impact on the validity of the conclusions, including inadequate problem specification, model misspecification errors, using biased proxies for target variables of interest, and using erroneous training data to create predictive models. In many cases, biases in the data may result from corresponding biases in human behavior and social policy. For example, an analysis of the New York Police Department's “stop and frisk” policy revealed that persons of African and Hispanic descent were stopped more frequently than whites, controlling for geographic factors and for race-specific estimates of crime participation.^6^ An algorithmic model trained on such data may reinforce an undesirable status quo.

In the health care setting, these challenges, if not adequately addressed, may impede health equity. For example, an algorithm trained on data from a nonrepresentative patient population may fail to provide adequate predictions in other settings. Even if the data are representative, failure to account for heterogeneity within the patient population may lead to suboptimal predictions (and as a result, worse outcomes) for patients with unusual variants of a disease. Target variable bias may manifest if we fail to account for whether patients will comply with a given treatment regimen, measuring the benefits to adherent patients only. Finally, as we more precisely predict the risks and benefits of treatment for various conditions, there is a danger that we will preferentially direct limited health care resources to those subpopulations with the best cost/benefit trade-offs. This may lead to systematic biases in health care for minority groups—who might respond differently to treatments developed for the majority.

We can mitigate these potential sources of bias and inequity by designing algorithmic decision-making approaches that explicitly account and correct for these issues. To do so, we propose a multifaceted interdisciplinary approach to using big data analytics in health care that combines insights from data scientists and domain experts:

(1) When evaluating the performance of a predictive model or decision-making algorithm in a real-world health care setting, it is insufficient to measure and compare algorithms in terms of overall prediction accuracy. Instead, we must explicitly measure equity criteria such as how false positives and false negatives are distributed among demographic groups, and the proportions of each group that are assigned a treatment.
(2) In designing algorithms for health care, data scientists should explicitly include fairness criteria as part of the model and objective function to be optimized. Doing so will improve algorithmic performance on the expanded set of aforementioned evaluation criteria, reducing disparities in care and in outcomes.
(3) Emphasis should be placed on advancing approaches for discovering heterogeneous treatment effects in health care data, identifying the relevant sources of variation within the patient population, and then accounting for these in an equitable way when making treatment decisions.
(4) Whenever possible, algorithms should be transparent—that is, it should be clear how the algorithm makes predictions or decisions—so that a domain expert can look at the underlying model, identify its assumptions and decide whether they are valid, and anticipate and correct failure modes that the developer did not consider. In addition, the data set(s) used to train the predictive model should be clearly described, so that the representativeness of the sample population and any systematic biases that might impact the model predictions can be assessed.
(5) Use of algorithms that lack transparency should depend on an external audit of the algorithm's outputs—for example, its predictions for a large patient population—to reveal whether systematic biases exist, and if so, to correct these biases. Algorithmic audits should be based on the combined input of human domain experts as well as automated approaches for bias detection.^7^

We believe that the systematic application of these five criteria to design, analyze, and evaluate algorithms used for health care decision-making has great potential to ensure that the deployment of such algorithms leads to the reduction, rather than exacerbation, of health disparities.

We are on the verge of massive health care transformation, which is in large part driven by big data and the innovations they inspire. We cannot afford to underestimate the unintended consequences of these forces on health care equality.

## Abbreviation Used

COMPAS: Correctional Offender Management Profiling for Alternative Sanctions
